# Whole-Genome Analysis of Extensively Drug-Resistant *Enterobacter hormaechei* Isolated from a Patient with Non-Hodgkin’s Lymphoma

**DOI:** 10.3390/genes15060814

**Published:** 2024-06-20

**Authors:** Cristina Motta Ferreira, Felipe Gomes Naveca, Guilherme Motta Antunes Ferreira, Maria de Nazaré Saunier Barbosa, Victor Costa de Souza, Franceline Oliveira Calheiros, Vander Silva Souza, William Antunes Ferreira

**Affiliations:** 1 Fundação Hospitalar de Hematologia e Hemoterapia do Amazonas—HEMOAM, Av. Constantino Nery, 4397, Chapada, Manaus 69050-001, Amazonas, Brazil; 2 Instituto Leônidas e Maria Deane—FIOCRUZ, Rua Teresina, 476, Adrianópolis, Manaus 69027-070, Amazonas, Brazil; 3 Programa de Pós-Graduação em Hematologia, Universidade do Estado do Amazonas—PPGH-UEA/HEMOAM, Av. Constantino Nery, 4397, Chapada, Manaus 69050-001, Amazonas, Brazil; 4 Fundação de Dermatologia Tropical e Venereologia Alfredo da Matta—FUAM, Rua Codajás, 24, Cachoeirinha, Manaus 69065-130, Amazonas, Brazil; wianfe@yahoo.com.br

**Keywords:** mutations, β-lactamases, efflux pump, plasmid, *ampC*, blood infections

## Abstract

Background: Currently, the *Enterobacteriaceae* species are responsible for a variety of serious infections and are already considered a global public health problem, especially in underdeveloped countries, where surveillance and monitoring programs are still scarce and limited. Analyses were performed on the complete genome of an extensively antibiotic-resistant strain of *Enterobater hormaechei*, which was isolated from a patient with non-Hodgkin’s lymphoma, who had been admitted to a hospital in the city of Manaus, Brazil. Methods: Phenotypical identification and susceptibility tests were performed in automated equipment. Total DNA extraction was performed using the PureLink genomic DNA mini-Kit. The genomic DNA library was prepared with Illumina Microbial Amplicon Prep and sequenced in the MiSeq Illumina Platform. The assembly of the whole-genome and individual analyses of specific resistance genes extracted were carried out using online tools and the Geneious Prime software. Results: The analyses identified an extensively resistant ST90 clone of *E. hormaechei* carrying different genes, including *bla*_CTX-M-15_, *bla*_GES-2_, *bla*_TEM-1A_, *bla*_ACT-15_, *bla*_OXA-1_ and *bla*_NDM-1_, [*aac(3)-IIa, aac(6′)-Ian, ant(2″)-Ia*], [*aac(6′)-Ib-cr*, (qnrB1)], *dfrA25*, *sul1* and *sul2*, *catB3*, *fosA*, and *qnr*B, in addition to resistance to chlorhexidine, which is widely used in patient antisepsis. Conclusions: These findings highlight the need for actions to control and monitor these pathogens in the hospital environment.

## 1. Introduction

Hospital-acquired infections continue to be a serious public health problem worldwide. According to the United States Centers for Disease Control and Prevention (CDC-Atlanta, GA, USA), approximately 1.7 million patients are affected by these infections while they are being treated for other health problems, and approximately 98 thousand of them (1:17) die [1,2,3].

The *Enterobacter cloacae* complex (ECC) is a common hospital pathogen responsible for different infections in humans [4]. ECC shows genomic heterogeneity and currently comprises seven species: *E. cloacae, E. hormaechei, E. asburiae, E. kobei, E. nimipressuralis, E. mori*, and *E. carcinogenus,* of which *E. cloacae* and *E. hormaechei* are the most [5,6,7] important emerging human pathogens [8]. The ECC is considered an important member of the *Enterobacteriaceae* family and is widely distributed in the environment and in the intestinal microbiota of humans and animals [6,7,9]. As an opportunistic pathogen, it can cause different types of infections such as pneumonia, bacteremia, urinary tract infections, skin infections, meningitis, brain abscess, and endocarditis [6,10]. 

These infections can be especially dangerous and even fatal when they occur in immunocompromised individuals or patients such as neonates and those diagnosed with hematological diseases such as leukemia, diabetes mellitus, burns, or multiple traumas, especially those admitted to an intensive care unit (ICU) [6,7,11,12]. The World Health Organization (WHO) classifies the ECC carbapenemase enzyme producers, and/or extended spectrum β-lactamase (ESβL) as PRIORITY 1: CRITICAL [13], and some strains of this complex, belonging to sequence type (ST) ST171 and ST78, are considered high-risk disseminators clones of important resistance genes [5].

Generally, the ECC is associated with multidrug resistance (MDR) phenotypes due to their ability to adapt to the hospital environment, their ability to acquire resistance genes through mobile genetic elements and their behavior as opportunistic pathogens. Also, the resistance mechanisms to cephalosporins in *Enterobacter* spp. to that are first-to third generation include the production of chromosomal AmpC β-lactamases [9,11]; the expression of EsβLs [9,11,12], and the expression of SHV-12 β-lactamase, which contributes to the resistance or reduced susceptibility to the third and fourth generation [9]. The *bla*_CTX-M-15_ gene is the most common in different species of ECC found in clinical samples [11,12], which, when associated with other resistance mechanisms and to aminoglycosides, quinolones, carbapenems, and colistin, represent a serious health public problem due to the high mortality rate as a result of the difficulty involved in their treatment [12]. 

The prevalence of carbapenem-resistant *Enterobacteriaceae* (CRE) has been increasing since the year 2000, and the mechanism involved in this resistance is due to the constitutive overexpression of AmpC β-lactamases, permeability of membrane (decrease in or loss of the outer membrane proteins OmpF and OmpC), and efflux pumps or plasmid, which encode carbapenem genes. The main mechanism of carbapenem resistance in the ECC is through plasmid transfer [6,9,14]. The genes that encode these enzymes are either of chromosomal origin or released by other transferable genetic elements, such as plasmids, transposons, and integrons [15]. 

Clinical and genomic studies reveal that the ECC demonstrates great ease in acquiring genes that encode resistance to broad-spectrum antibiotics, including several carbapenemase genes [2,16,17], thus facilitating the dissemination of high-risk clones (STs) of global distribution [2] responsible for infections with a mortality rate of up to 44% [12,14,18]. 

Therefore, due to these characteristics of resistance to different antibiotics, carbapenem-resistant *Enterobacter* spp. currently constitutes a serious health problem for patients, especially those who are immunocompromised and, due to the risks of dissemination in a hospital environment, they have come to be considered an enormous challenge for both effective antibacterial therapy and control of infectious processes [12,14,19]. 

The study of the bacterial genome has provided extremely relevant information for the scientific community. The knowledge about the various data related to bacterial distribution, genomic diversity, DNA mutations, identification of species, genetic modifications, and genetic profile related to antimicrobial resistance favors different global epidemiological surveillance studies and new taxonomic classifications [19,20]. In this study, we describe the analyses carried out after the genome sequencing of an extensively resistant strain of *E. hormaechei*, isolated from the urine culture of a patient with non-Hodgkin’s lymphoma, treated at the Fundação Hospitalar de Hematologia e Hemoterapia do Amazonas (FHEMOAM), a blood center in the city of Manaus, Brazil. 

## 2. Materials and Methods

The procedures for the microbiological diagnosis of the patient urinary tract infection caused by *E. hormaechei* described in this report were carried out at the FHEMOAM Clinical Microbiology Laboratory, with samples being collected during routine procedures, while the next-generation sequencing (NGS) protocols of the *E. hormaechei* bacteria were carried out in collaboration with the Molecular Biology Laboratory of the Instituto Leônidas and Maria Deane—FIOCRUZ da Amazônia. 

### 2.1. Cultivation, Identification, and Susceptibility Testing

The biological sample provided to the bacteriology laboratory was urine that was cultured immediately on URILAB-CLED/MacConkey slide culture media (Laborclin—Biokar Diagnostics, Allonne, France) for primary seeding. After incubation for 24 h and obtaining a colony count result of 1,000,000 CFU/mL, the subculture was carried out in 5% sheep blood agar and MacConkey agar (Himedia, Hexasystems, Mumbai, India), with another round of incubation for more 24 h at 35.4 °C. A positive and pure colony grown on MacConkey agar was selected for subsequent tests and Gram staining. Using standard microbiological procedures, phenotypic identification and susceptibility testing for antimicrobials, with the respective minimum inhibitory concentration (MIC) values were performed using automated equipment (VITEK-2, bioMerieux, Craponne, France), according to the manufacturer’s instructions and Brazilian Committee on Antimicrobial Susceptibility Testing—BrCAST and Clinical & Laboratory Standards Institute—CLSI, 2023 guidelines. The *Escherichia coli* ATCC 25,922 strain was used as a quality control for susceptibility testing. The *E. hormaechei* aliquot was stored at −80 °C in a cryovial with brain heart infusion broth (Himedia, Hexasystens-Mumbai, India) + 20% glycerol for subsequent molecular testing.

### 2.2. Genomic DNA Extraction

Total DNA extraction was performed using the PureLink genomic DNA mini-Kit (Invitrogen, Carlsbad, CA, USA) and quantified using a spectrophotometer (Nanodrop 1000 ThermoFisher, Waltham, MA, USA), according to the manufacturer’s instructions.

### 2.3. Whole Genome Sequencing, MLST, and Resistance Genes

The genomic DNA library was prepared with the Illumina Microbial Amplicon Prep kit (iMAP) (https://www.illumina.com/products/by-type/sequencing-kits/library-prep-kits/microbial-amplicon-prep.html, accessed on 29 April 2024) and sequenced in the MiSeq Illumina Platform (Illumina, San Diego, CA, USA), configured to generate paired-end reads of 150 bp according to the standard protocol, and the assembly of the whole-genome and individual analyses of specific resistance genes extracted from the genome were carried out using software, such as Geneious Prime v. 2024, to compare the genome with the sequences deposited in GenBank—National Library of Medicine (NIH) (https://www.ncbi.nlm.nih.gov/genbank/, accessed on 10 May 2024). 

Another analysis of the genome was performed via online services, such as the Center for Genomic Epidemiology (https://www.genomicepidemiology.org/, accessed on 10 May 2024), to investigate plasmid multi-locus sequence typing (pMLST 2.0) and MLST profile (MLST-2.0 Server), the presence of antibiotic- and disinfectant-resistant genes (Kmer resistance 2.2) and (ResFinder 4.4.2), virulence genes (VirulenceFinder 2.0), plasmids (PlasmidFinder 2.0), mobile genetic elements (mobileElementFinder v. 1.0.3), and the Bacterial and Viral Bioinformatics Resource Center to verify the comprehensive genome via the analysis service—PATRIC (https://www.bv-brc.org/, accessed on 10 May 2024).

### 2.4. Nucleotide Sequence Accession Number

The data generate in this study are available in the National Library of Medicine repository, NCBI database, under accession number: JBBMKH000000000.1, https://www.ncbi.nlm.nih.gov/genbank/ accessed on 10 May 2024.

### 2.5. Patient Data

A male patient, 63 years old, from the rural area of the municipality of Itacoatiara (Arari River), Amazonas, where he lived in a wooden house with an external bathroom and septic tank, with his two dogs as well as birds (chickens and ducks) and some cattle. He worked as a farmer and as a captain of the boat for the SOS Ribeirinho service in the community where he lived. During a consultation at the health unit in the city near where he lived, he denied being a smoker; however, he reported having hypertension for over 10 years.

The medical records showed that in March 2021, he contracted COVID-19. In a new consultation in December 2022, he presented dizziness, the presence of nodules in the inguinal region, and weight loss of approximately 10 kg in 30 days and reported to be in a feverish state, albeit without proper measurement, as well as having dyspnea and chest pain. In February 2023, he was admitted to the emergency room close to the rural community where he lived.

A blood count was performed which showed anemia, while the urine test (ASE) showed proteinuria, leukocyturia (13 per field), hematuria (6 per field), and moderate bacterial flora; the fecal parasitological examination (FPE) revealed the presence of *Entamoeba histolytica* and *Endolimax nana*. In March 2023, he traveled to Manaus (capital of the state of Amazonas) for consultation at the FHEMOAM, where he was diagnosed with non-Hodgkin’s lymphoma. In the same month, he began treatment with oral corticosteroids (predinisone) 100 mg/day for 5 days + cyclophosphamide 750 mg IV for 1 day (non-specific chemotherapy cycle).

He developed persistent edema of the lower limbs (lower limbs) and underwent a urine culture with a positive result for *E. hormaechei*, and, at this point, he started infusion with meropenem 6 g/day and vancomycin 2 g/day. After 16 days of hospitalization at FHEMOAM, his clinical condition worsened with dyspnea, psychomotor disorientation, and non-responsiveness to verbal stimuli; underwent an indwelling bladder catheterization; and was subsequently transferred to a highly complex emergency hospital, when on 18 March 2023 he died, due to sepsis.

## 3. Results

The microbiological urine culture examination, which was carried out during the patient’s hospitalization at FHEMOAM, made it possible to identify an isolate of extensively resistant (XDR) *E. hormaechei*. The antimicrobial susceptibility test, repeated twice, and the respective MICs are shown in Table 1, which presents an isolate with a multidrug resistance profile and susceptibility only to colistin.

### Genotyping and Antimicrobial Resistance Genes 

The complete genome of *E. hormaechei* showed that it is composed of 159 contigs, with an estimated length of 5,144,869 bp and an average G+C content of 55.25%. The N50 length is 148,435 bp, and the L50 count is 32. The multi-locus sequence typing (MLST) analysis showed that *E. hormaechei* belongs to the ST90 sequence type.

The analyses also detected the presence of genes with resistance to β-lactams and other genes, such as [*aac(3)-IIa, aac(6′)-Ian, ant(2″)-Ia, aac(6′)-Ib-cr*] (aminoglycosides and quinolones), *dfrA25*(trimethoprim-sulfamethoxazole), *sul1* and *sul2* (sulfonamides), *catB3* (chloramphenicol), *qnr*B1, *fos*A (fosfomycins), *bla*_CTX-M-15_, *bla*_GES-2_, *bla*_TEM-1A,_ *bla*_OXA-1_, *bla*_NDM-1_, and *bl*a_ACT-15_, of which analysis using the reference sequence in GenBank AFU25653 showed a mutation at the position Ser164Pro (Table 2). 

Other genes, such as the disinfectant-resistant *qacE*; others involved in intrinsic antibiotic resistance (*macA* and *macB*); ones that mediate the stress antibiotic response (*marA* and *marB*); virulence *nlpI* (lipoprotein NlpI precursor) involved in the adhesion to and invasion of epithelial cells [21]; and ironN (enterobactin sidephore receptor protein) [22] were detected. As for plasmids, the analysis revealed the presence of *Inc*FIA (HI1), pKP1433, *Col* (pHAD28), and *Inc*C with a ST3 profile for *IncA*/C according to the pMLST analysis. 

In relation to the mobile genetic elements (MGEs) (mediate horizontal gene transfer), we also identified the insertion sequences: ISEc9 (family IS1380) with *bla*_CTX-M-15;_ IS6100 (family IS6) with *sul1*; Transposon Tn801 (family Tn3) with *bla*_TEM-1A_; ISEhe3 (IS3) with repA; ISKpn24 and cn_15018_ISKpn24 (IS66) (family IS66) with *Inc*FIA(HI1); ISAs29 (family IS21), ISKpn18 (family IS3), and ISSen4 with incA/C2; ISKpn26 (family IS5); ISEc33 (family IS630); IS5075 (family IS110); IS1006 (family IS6); and transposon *Tn*6196, involved in the carriage of resistance genes to β-lactamases and other resistance genes. Analyses carried out with genes extracted directly from the *E. hormaechei* genome and compared with sequences available at the National Center for Biotechnology Information (NCBI) made it possible to identify several mutations in protein sequences related to resistance to tigecycline (RamA, RamR, and AcrR), quinolones (GyrA and GyrB), and cephalosporins (AmpC, AmpR, and Blc) (Supplementary Material—Table S1). 

## 4. Discussion

The emergence of multidrug-resistant Gram-negative pathogens that produce β-lactamases (ESβL, AmpC, and carbapenemases) is currently considered a major public health problem. These pathogens are associated with hospital and community infections, with β-lactamases being a widely used determinant of resistance disseminated in mobile genetic elements, mainly enterobacteria, and opportunistic pathogens [9,15,17,19]. The World Health Organization [13] warns in its reports that the Enterobacteriaceae (including Escherichia coli, Klebsiella pneumonia, Serratia, and Proteus) EsβL-producing, carbapenem-resistant species, involved in infections such as urinary tract, pneumonia, and septicemia [21,22], are those that have a more significant phenotypic profile of resistance. The analysis of the *E. hormaechei* genome showed concordance with our antimicrobial susceptibility test results, in which we identified a resistance profile to antimicrobial classes such as aminoglycosides, quinolones, β-lactams, folate pathway antagonist, fosfomicin, macrolide, tetracyclines, amphenicol, polymyxin, and steroid antibacterial (fusidic acid). Furthermore, the analyses made it possible to identify other determinants of resistance, such as *bla*_CTX-M-15_, *bla*_GES-2_, *bla*_TEM-1A_, *bla*_OXA-1_, and, notably, *bla*_NDM-1_ and *bla*_ACT-15_, rare plasmid-encoded variants of the *amp*C gene, with few records in the literature [16,22], which presented a mutation in the Ser164Pro position of the *bla*_ACT-15_ gene protein.

We did not find other *amp*C variants genes, such as *bla*_ACT-59_ and *bla*_ACT-45_, but these have already been identified in strains of *E. cloacae* ST873 in China and in *E. hormaechei* in Argentina [8,22]. In another study, carried out in Spain, *bla*_ACT-12,_ *bla*_ACT-14,_ *bla*_ACT-15,_ *bla*_ACT-17,_ *bla*_ACT-18,_ *bla*_ACT-19_, and *bla*_ACT-22_ genes were also identified but in *E. cloacae* isolates with the *ampR* and *blc* genes [23]. In our study, we observed the *ampR* and *blc* genes in the isolates of *E. hormaechei*. The *ampR* is a master regulatory gene that switches the expression of genes on and off, including antibiotic resistance genes [24], while *blc* genes also play a role in the dissemination of antibiotic resistance genes and in the activation of immunity [24,25].

These enzymes, including plasmid-mediated and derepressed or hyperproduced chromosomal AmpCs, are of extreme importance in clinical treatments because they are active against all β-lactams (including cephamicins, a very efficient antibiotic against anaerobic agents), except fourth-generation cephalosporins and carbapenems. They are also often associated with multi-resistance genes such as those of resistance to aminoglycosides, quinolones, sulfonamides, trimethoprim, tetracycline, chloramphenicol, and other β-lactamase genes [26]. The broad-spectrum resistance profiles to β-lactams observed in *Enterobacteriaceae* are either due to the production of ESβL or mediated by the overexpression of the chromosomal *ampC* gene, which codes for cephalosporinase, with plasmids being the main carriers of multiple resistance genes, including ESBL, in clinical isolates, thus becoming important vehicles for horizontal transfer in this genus [27]. The association of β-lactamase resistance genes, *bla*_CTX-M-15_, *bla*_GES-2_, *bla*_TEM-1A_, *bla*_ACT-15_, *bla*_OXA-1_, and *bla*_NDM-1_, with other genes, such as [*aac(3)-IIa, aac(6′)-Ian, ant(2″)-Ia*], [*aac(6′)-Ib-cr*, (*qnr*B1)], *dfrA25*, *sul1* and *sul2*, *catB3*, *fosA*, in the same isolate, increasingly potentiate the multi-resistance profile to different antibiotics classes such as β-lactams, aminoglycosides, trimethoprim–sulfamethoxazole, tetracyclines, and amphenicol [16,18,20]. 

The analysis of the MLST showed that the *E. hormaechei* genotype belonged to ST90 but had a resistance profile. These results are in accordance with other studies that detected resistance genes, such as *bla*_CTX-M-15_, *bla*_SVH-12_, *bla*_TEM-1B,_ *bla*_ACT-9,_ *bla*_ACT-7_, and *bla*_OXA-1_, in addition to resistance to antibiotics such as trimethoprim, rifampicin, aminoglycosides, and fluoroquinolones, which have also been identified in bacterial isolates obtained from infectious processes in patients with or without hematological diseases who were admitted to hospitals [22,26,28].

The genome of the isolate analyzed also showed a resistance profile to ciprofloxacin with a MIC ≥ 4 µg/mL. Mutations have been identified in the GyrA and GyrB of target genes *gyrA* and *gyrB* but none in the region of ParC (Table S1), thus highlighting that resistance to these antibiotics is not always associated with mutations in ParC (Supplementary Material—Table S1). The resistance genes *aac(3)-IIa, aac(6′)-Ian, ant(2″)-Ia*, and *aac(6′)-Ib-cr* were identified, which are related to the induction of quinolone and aminoglycoside resistance, as well as the common efflux pump genes, *marA, ramA*, and *AcrAB*, of which their overexpression decreases the cellular concentration of the drug [22,29,30,31]. The resistance to carbapenems observed in this study may be due to the constitutive overexpression of AmpC combined with the interruption of membrane permeability, the acquisition of plasmid-encoded carbapenemase genes by conjugation or transformation, self-medication or non-compliance with the dosage, admissions to other hospitals, prolongation of the disease, or the presence of class I integron [32]. However, chromosomally encoded genes are rarer [32,33,34], thus causing great difficulty for clinicians when it comes to therapeutic options [16]. 

Regarding resistance to tigecycline, observed in the susceptibility profile of this study, genome analysis revealed the presence of the multidrug resistance efflux pump (MDR) gene *acrAB*. Changes such as mutations in efflux proteins, ribosomal protection proteins, enzyme inactivation (*tet* (A), *tet* (M), and *tet* (X)), or the overexpression of the *AcrAB*-TolC and *OqxAB* efflux pumps contribute fundamentally to the multidrug resistance process [30,35].

Generally, the efflux pump is regulated by the *ramR* and *acr* genes, and mutations or deletions in the gene *ramR* elevate *ramA* and *acrAB* gene expression, favoring the multidrug resistance phenotype, including cross-resistance with tetracyclines, β-lactams, and chloramphenicol [29,36], arising from the broad substrate specificity that the *AcrAB*-TolC pump can eliminate [29]. Furthermore, genes involved in the regulation of the efflux pump and porin expression may also contribute to the adaptation of strains to their environment and the evasion of the host’s immune system [37].

Another gene identified in *E. hormaechei* was *qacE*, which is related to resistance to the following compounds: benzalkonium chloride, ethidium bromide, cetylpyridinium chloride, and chlorhexidine. This is another factor of great clinical concern since products containing some of these compounds are used as mouthwashes [31], disinfectants for processing products, cleaning environments, and disinfecting skin to collect biological material. In this context and given the diversity of onco-hematological diseases treated at FHEMOAM, it is a fact that patients during medical and dental care make use of these antiseptic agents and that they are widely used in the most diverse health centers and hospitals in the region.

Regarding the plasmids identified in our analyses, as well as in other studies, the replicons Col (pHAD28), *Inc*FIA(HI1), pKP1433, and *Inc*C are commonly observed in isolates from the *Enterobacteriaceae* family, carrying resistance genes to the third/fourth generation of the cephalosporin group of antibiotics associated with co-resistance to other classes of antibiotics [30,38]. 

In addition to the analyses regarding extensive antibiotic resistance, the presence of the ST90 clonal lineage, certainly in circulation in the region, is an important aspect in the context of molecular epidemiology. This ST was also classified in China as carrying the *bla*_NDM-1_ gene associated with colistin resistance gene *mcr*-9 [6], while in Denmark, the *bla*_NDM-1_ gene was associated with *bla*_OXA-436_ (*bla*_OXA-48_-like), with its suspected origin of contamination being an environmental reservoir [39]. 

In the United Kingdom, the epidemic, hypervirulent, and lethal clonal strains of *E. hormaechei*, such as ST133, were isolated from blood cultures [40]. Another study also identified clonal strains such as ST133 and ST418 from *E. hormaechei*, obtained from blood cultures, with ST133 being considered the most hypervirulent and carrying the carbapenemase resistance gene and *mcr*-9 [41]. In Croatia, an outbreak of *E. hormaechei* ST133 carrying *bla*_NDM-1_ occurred in the intensive care unit (ICU), with two deaths due to severe infection [41], while in Germany, in the ICU, different subspecies of *E. hormaechei* were identified arising from infectious processes. In the city of Guadalupe, clones such as ST114 and ST1503 of *E. xiangfangensis* were the most prevalent isolates in clinical samples [27]. 

In Franconia, a province of Bavaria, a series of five cases of blood infections caused by *E. hormaechei* were described. These were identified in the neonatal intensive care units (NICU) of a university hospital and led to the death of three patients [42], while in Brazil, there is only one report in the literature of the occurrence of the *E hormaechei* ST90 clonal strain being isolated from soil [43]. Other studies report only cases of blood infection in neonates, in three NICU, with a complete cure being the outcome [44]. This indicates that *E. hormaechei* is the most prevalent species among clinical isolates of the ECC complex [20], which is responsible for 65–75% of the hospital infections worldwide [45] and has thus emerged as an extremely relevant hospital pathogen. Both blood infections and colonization caused by these pathogens can be facilitated by environmental contamination and the relaxation of hospital infection control practices among health professionals [46,47] because this constant exposure makes them easily colonized by multidrug-resistant bacteria on the skin or in the nose and thus are important transmission vectors and sources of hospital outbreaks. Therefore, due to the ease of the adaptation of *E. hormaechei* to the hospital environment, serving as a reservoir for the transmission of antibiotic resistance genes in hospital infections [45], this species is a pathogen of great threat to human health, requiring the adoption and constant monitoring of preventive measures and patient management in order to avoid outbreaks of hospital infections.

## 5. Conclusions

We identified the extensively resistant *E. hormaechei* ST90 clone isolated from a non-Hodgkin’s lymphoma patient undergoing treatment at Fundação HEMOAM carrying the *bla*_CTX-M-15_, *bla*_GES-2_, *bla*_TEM-1A_, *bla*_ACT-15_, *bla*_OXA-1_, and *bla*_NDM-1_ genes_,_ together with [*aac(3)-IIa, aac(6′)-Ian, ant(2″)-Ia*], [*aac(6′)-Ib-cr*, (qnrB1)], *dfrA25*, *sul1* and *sul2*, *catB3*, *fosA*, and *qnr*B genes, this being the first report in the northern region of Brazil. Genome studies are important for investigating and understanding bacterial resistance mechanisms, especially in the hospital environment, as the management of patients infected by these types of pathogens is difficult due to the extremely limited number of therapeutic options.

## Figures and Tables

**Table 1:** *E. hormaechei* antimicrobial susceptibility test.

Antibiotic Class	Antibiotics	MIC ^4^ (mg/L)	Interpretation
β-Lactams: (penicillins ^1^; carbapenens ^2^; cephalosporins ^3^)	Ampicillin ^1^	≥32	R ^5^
	Ampicillin/sulbactam ^1^	≥32	R
	Piperacillin/tazobactam ^1^	≥128	R
	Ertapenem ^2^	≥8	R
	Meropenem ^2^	≥16	R
	Imipenem ^2^	≥16	R
	Cefuroxime ^3^	≥64	R
	Axetil cefuroxime ^3^	≥64	R
	Cefoxitin ^3^	≥64	R
	Ceftazidime ^3^	≥64	R
	Ceftriaxone ^3^	≥64	R
	Cefepime ^3^	≥64	R
Aminoglycosides	Amikacin	≥64	R
	Gentamicin	≥16	R
Fluoroquinolone	Ciprofloxacin	≥4	R
Glycylcyclines	Tigecycline	≥8	R
Polymyxins	Colistin	≤0.5	S ^6^

^1^ = Penicillins; ^2^ = Carbapenens; ^3^ = Cephalosporins; ^4^ = Minimum inhibitory concentration; ^5^ = resistant; ^6^ = suscetible.

**Table 2:** Antimicrobial resistance genes.

AMR Mechanism	Genes
**Antibiotic activation enzyme**	*KatG*
**Antibiotic inactivation enzyme**	*aac(3)-II, III, IV, VI, VIII, IX, X, aac(6′)-Ib/aac(6′)-II,* ACT/MIR family, CatB family, CTX-M family, GES family, NDM family, OXA-1family, TEM family
**Antibiotic resistance gene cluster, cassette, or operon**	*MarA, MarB, MarR*
**Antibiotic target susceptible species**	*Alr, Ddl, dxr, EF-G, EF-Tu, folA, Dfr, folP, gyrA, gyrB, inha, fabI, Iso-tRNA, kasA, MurA, rho, rpoB, rpoC, S10p, S12p*
**Antibiotic target protection protein**	*BcrC, QnrB*10
**Antibiotic target replacement protein**	*fabV*
**Efflux pump conferring antibiotic resistance**	*AcrAB*-TolC, *AcrAD*-TolC, *AcrEF*-TolC, *AcrZ, EmrAB*-TolC, *EmrD, MacA, MacB, MdfA/Cmr, MdtABC*-TolC, *MdtL, QacE, SugE,* TolC/OpmH
**Gene conferring resistance via absence**	*gidB*
**Protein altering cell wall charge conferring antibiotic resistance**	GdpD, PgsA
**Protein modulating permeability to antibiotic**	OccD6/OprQ
**Regulator modulating expression of antibiotic resistance genes**	*AcrAB*-TolC, *EmrAB*-TolC, *H-NS, OxyR*

Source: Bacterial and Viral Bioinformatics Resource Center.

## Data Availability

The datasets generated and/or analyzed during the current study are available in the National Library of Medicine repository, NCBI database, under accession number: JBBMKH000000000.1, https://www.ncbi.nlm.nih.gov/genbank/.

## Supplementary Materials

The following supporting information can be downloaded at: https://www.mdpi.com/article/10.3390/genes15060814/s1, Table S1: Mutations observed in the protein sequences of some determinants of resistance to quinolones and tigecycline and cephalosporins.
