# Supplementary material for: Whole-Genome Analysis of Extensively Drug-Resistant Enterobacter hormaechei Isolated from a Patient with Non-Hodgkin’s Lymphoma
Source: Genes (Basel). 2024 Jun 20;15(6):814. doi: 10.3390/genes15060814 (PMC11202416; doi:10.3390/genes15060814)
Supplement: Supplementary file 1 [file genes-15-00814-s001.zip › genes-3054672-supplementary.pdf]

Supplementary Material-Table S1

Mutations observed in the protein sequences of some determinants of resistance to quinolones and tigecycline and cephalosporins

Antibiotics	Protein	Mutations	GenBank Accession
Quinolone[1]	GyrA	Thr408Ala, Ala409Ser, Ile411Val, Ser412Ala, Ile509Val, Ala526Ser, Gln536Arg, Lys691Ser, Asp709Asn, Ile737Val, Gy759Ser, Asp861Glu, Val 866Ala, Asp871Glu, Ala872Val	WP_013098009.1
	GyrB	Thr682Ala, Ala691Thr, Glu151Asp, Glu161Asp	STP60708.1
Tigecycline	RamA	Ile18Val, Leu73Met, Ala83Glu, Ser121Gly	AFK13828
	RamR	Ser31Ala, Ala32Leu, Ser36Asn, Glu53Asp,Glu57Ala, Ala61His, Ile62Leu, Leu64Gln, Arg65Asp,Val67Cys, Arg68Gln, Ile71Leu, Gly73Asn, Pro76Arg, Asp77Thr, Glu78Ile, Lys79Thr, Arg80Glu, Asn84His, Ala85Thr, Ile90Val, Val97Ile, Met101Leu, Glu102Ala, Lys104Ala, Arg108Gln, Met109Ile, Ala110Gly, Leu11Val, Arg114Lys, Ile115Leu, Thr116Asn, Asp117Ala, Arg120Glu, Arg121Gln, Gln122Ala, Glu125Asp, Ser126Met, Asn131His, Met133Leu, Gln135Arg, Leu136Arg, Val138Ile, Lys139Arg, Glu140Pro, Ile141Val, Leu143Met, Glu145Asp, Ala146Glu, Tyr147Phe, Arg148Lys, Ala149Thr, Leu154Met, Ile163Met, Ser167Ala, His168Arg, Gln171Ser, Arg174Val, Glu175Asp, Ile176Phe,Ile177Lys, His186Arg, Ala187Gly, His189Thr, Ala191Glu, Ala193Asn	ASU06826
	AcrR	Asp196Glu, His212Leu, His213Ser	WMA67388
Cephalosporin	AmpC	Thr4Lys, Ser14Gly, Thr15Ile, Val19Ala, Met24Val, Glu34Ala,Arg35Asn, Ala45Ser, Ile46Val, Glu56Gln, Gln58Lys, Phe62tyr, Val69Ile, Gly107Asp, Lys145Arg, Glu149Gln, Lys147Thr, Ser190Pro, Ile195Met, Phe200Leu, Is231Arg, Glu240Gln, Ser253Asn, Val257Ala, Lys260Ala Asp262Glu, Ser263Asn, Leu264Val, Gln265Ala, Asn267Ala, Arg270Lys, Lys271Gln, Leu273Ile, Thr274Ala, Val283Ile, Ala285Ser, Asp300Glu, Lys302Asn, Asn310Ser, Ala319Val, Arg320Val, Asn330Lys, Leu355Ile, Lys363Thr, Arg376His, Ser379Glu	ADX96007
	AmpR	Ala43Thr, Ser89Asn, Gln90His, Leu112Gln, Ala114Glu, Cys119Gly, Asp156Glu, Pro162His, Ser166Ala, Glu172Asp, Ser175Ala, Val176Ser, Lys178His, Lys185Arg, Ala198Thr, Ser208Pro, Ser247Ala, Asp260Glu, Ser273Ala, Ala282Ser, Gln283Arg, Gly287Glu, Gln290Lys	ADX96006
	Blc	Pro33Asn, Leu49Met, Arg52Gln, Gln55Arg, Ala62Val, Asn63Ser, Pro66Ala, Gln73Arg, Glu84Gln, Ile90Val, Lys92Gln, Asp98Ala, Pro99Ser, Arg100Asn, Leu104Met, Phe109Ile, Asp136Asn, Ile140Leu, Ser149Thr, Val158Ile, Ser167Thr, Pro175Leu	ADX96008

1 = No mutations were observed in ParC
